# Nitric oxide synthase polymorphisms, gene expression and lung function in chronic obstructive pulmonary disease

**DOI:** 10.1186/1471-2466-13-64

**Published:** 2013-11-06

**Authors:** Farzian Aminuddin, Tillie-Louise Hackett, Dorota Stefanowicz, Aabida Saferali, Peter D Paré, Amund Gulsvik, Per Bakke, Michael H Cho, Augusto Litonjua, David A Lomas, Wayne H Anderson, Terri H Beaty, Edwin K Silverman, Andrew J Sandford

**Affiliations:** 1 The University of British Columbia James Hogg Research Centre, Providence Heart + Lung Institute, Vancouver, Canada; 2 Haukeland University Hospital and Institute of Medicine, University of Bergen, Bergen, Norway; 3 Channing Laboratory and Pulmonary and Critical Care Medicine, Department of Medicine, Brigham and Women’s Hospital and Harvard Medical School, Boston, MA, USA; 4 Cambridge Institute for Medical Research, University of Cambridge, Cambridge, UK; 5 GlaxoSmithKline Research and Development, Research Triangle Park, South of Durham, NC, USA; 6 Johns Hopkins School of Public Health, Baltimore, MD, USA

**Keywords:** Chronic obstructive pulmonary disease, Nitric oxide synthase, Polymorphism, Gene expression

## Abstract

**Background:**

Due to the pleiotropic effects of nitric oxide (NO) within the lungs, it is likely that NO is a significant factor in the pathogenesis of chronic obstructive pulmonary disease (COPD). The aim of this study was to test for association between single nucleotide polymorphisms (SNPs) in three NO synthase (NOS) genes and lung function, as well as to examine gene expression and protein levels in relation to the genetic variation.

**Methods:**

One SNP in each NOS gene (neuronal NOS (*NOS1*), inducible NOS (*NOS2*), and endothelial NOS (*NOS3*)) was genotyped in the Lung Health Study (LHS) and correlated with lung function. One SNP (rs1800779) was also analyzed for association with COPD and lung function in four COPD case–control populations. Lung tissue expression of *NOS3* mRNA and protein was tested in individuals of known genotype for rs1800779. Immunohistochemistry of lung tissue was used to localize NOS3 expression.

**Results:**

For the *NOS3* rs1800779 SNP, the baseline forced expiratory volume in one second in the LHS was significantly higher in the combined AG + GG genotypic groups compared with the AA genotypic group. Gene expression and protein levels in lung tissue were significantly lower in subjects with the AG + GG genotypes than in AA subjects. NOS3 protein was expressed in the airway epithelium and subjects with the AA genotype demonstrated higher NOS3 expression compared with AG and GG individuals. However, we were not able to replicate the associations with COPD or lung function in the other COPD study groups.

**Conclusions:**

Variants in the NOS genes were not associated with lung function or COPD status. However, the G allele of rs1800779 resulted in a decrease of *NOS3* gene expression and protein levels and this has implications for the numerous disease states that have been associated with this polymorphism.

## Background

Nitric oxide (NO) is a molecule that is involved in many physiological and pathological pathways and can have either beneficial or detrimental effects. Many studies have shown that NO has protective effects on human airways such as muscle relaxation, attenuation of airway hyper-responsiveness to bronchoconstrictor stimuli, and the killing of invading microorganisms [1]. In contrast, adverse effects of NO have also been observed, such as vasodilation of the bronchial circulation, increased airway secretions and the promotion of pro-inflammatory pathways, as well as necrosis and apoptosis [1].

Endogenous NO is primarily synthesized by enzymes known as NO synthases (NOS) which catalyze the cellular production of NO from arginine. There are three known NOS isoforms: neuronal NOS (NOS1), inducible NOS (NOS2), and endothelial NOS (NOS3). In humans, NOS1 can be found in neurons and endothelial cells in the lung [2], while NOS3 is found in bronchiolar epithelial cells and the endothelium [3,4]. NOS2 is expressed in the human airway epithelium [5], lung endothelium [2], and alveolar macrophages [2].

Due to its pleiotropic effects, it is likely that NO is a significant factor in the pathogenesis of lung diseases such as chronic obstructive pulmonary disease (COPD), which is characterized by airflow limitation that is not fully reversible. There is evidence to suggest that NOS genes are associated with COPD. Recently, the mRNA and protein expression of *NOS1* and *NOS2* were observed to be increased in the peripheral lung tissue of smokers with COPD compared with nonsmoker controls, whereas the opposite effect was detected for *NOS3* expression [6]. Another study reported that the numbers of NOS2^+^ and NOS3^+^ cells were increased in the bronchial submucosa of smokers with COPD compared with nonsmoker controls [7]. Furthermore, deficiency of NOS2 has been shown to be protective against cigarette smoke-induced emphysema in a mouse model [8].

To further determine the effects of NOS in COPD, it is important to determine whether single nucleotide polymorphisms (SNPs) in NOS genes are associated with phenotypes related to the disease. It has been widely acknowledged that genetic factors account for some of the variability of lung function among smokers [9,10], suggesting an interaction between genetic and environmental influences on disease progression. The aim of this study was to determine whether NOS gene variants were associated with phenotypes related to COPD. We examined the rate of decline of lung function and baseline lung function in smokers with mild to moderate airflow obstruction from the Lung Health Study (LHS) in relation to polymorphisms in three NOS genes. The LHS was a randomized trial of an anti-smoking intervention and bronchodilator treatment in volunteer smokers [11]. We selected polymorphisms in NOS genes that had previously been associated with gene function or COPD-related traits [12-14]. We sought to determine whether these polymorphisms were associated with lung function decline and baseline level in COPD patients in the LHS as well as with COPD and lung function in four replication case–control sets.

## Methods

### Ethics statement

The investigation of the LHS and lung tissue samples was approved by the University of British Columbia/Providence Health Care Research Ethics Board and all subjects provided written informed consent. We attempted to replicate the associations in subjects from the following previously recruited populations: Norway COPD Cohort (GenKOLS) [15], National Emphysema Treatment Trial (NETT) [16,17], Normative Aging Study (NAS) [18], Evaluation of COPD Longitudinally to Identify Predictive Surrogate Endpoints (ECLIPSE) [19] and COPDGene [20]. These studies were approved by the relevant institutional review boards and all subjects provided written informed consent. For the NAS, anonymized data were used, as approved by the institutional review boards of Partners Healthcare System and the Boston VA.

### Study participants

The participants in the primary analysis were from the National Heart, Lung, and Blood Institute sponsored LHS cohort [11], consisting of smokers who had mild/moderate lung function impairment at the start of the study. Table 1 provides the characteristics of the LHS participants. Of the 5887 total participants in the LHS cohort, 4132 individuals of Caucasian descent had DNA samples available for the study. Lung function at the start of the study was expressed as forced expiratory volume in 1 second (FEV_1_) as a percentage of predicted value. The change in lung function, measured as change in FEV_1_ % predicted per year over a five-year period, was also an outcome measure of this study. For gene expression in lung tissue, genomic DNA, mRNA and protein were isolated from lung tissue from Caucasian patients who had undergone lobar or lung resection surgery for a localized lung cancer (n = 27). These samples were obtained from the James Hogg Research Centre Lung Registry. SNPs that showed association with lung function in the LHS cohort were genotyped in four Caucasian case–control cohorts: 1) the first 1000 COPDGene study subjects, 2) the COPD cases and smoking controls of the ECLIPSE study, 3) the NETT cases and NAS controls, and 4) the GenKOLS case–control population from Norway (Table 2) as described previously [21].

**Table 1:** **Characteristics of the 4132 Lung Health Study participants** (**2611 male**, **1521 female**)

**Characteristic**	**Total**
Age (mean ± SD), years	48.5 ± 6.7
Smoking history (mean ± SD), pack-years^a^	40.4 ± 18.4
Baseline FEV_1_ post-bronchodilator (mean ± SD), % predicted^b^	78.55 ± 9.10
FEV_1_ post-bronchodilator rate of decline (mean ± SD), % predicted / year^c^	-0.97 ± 1.78

^a^Number packs of cigarettes smoked per day × number of years of smoking.

^b^Lung function at the start of the study measured as forced expiratory volume in 1 second (*FEV*_1_).

^c^Change in lung function over a five-year period measured as forced expiratory volume in 1 second (*FEV*_1_).

**Table 2:** **Characteristics of the case**–**control replication study groups**

**Characteristic**	**COPDGene**		**ECLIPSE**		**NETT/NAS**		**GenKOLS**	
	**Cases**	**Controls**	**Cases**	**Controls**	**Cases**	**Controls**	**Cases**	**Controls**
Number of participants	499	501	1764	178	373	435	863	808
Age (mean ± SD), years	64.77 ± 8.12	60.20 ± 8.66	63.63 ± 7.10	57.48 ± 9.44	67.47 ± 5.78	69.8 ± 7.49	65.53 ± 10.03	55.62 ± 9.71
Smoking history (mean ± SD), pack-years^a^	54.76 ± 26.69	38.87 ± 21.07	50.29 ± 27.42	32.11 ± 24.84	66.43 ± 30.68	40.66 ± 27.85	31.98 ± 18.46	19.66 ± 13.58
FEV_1_ (mean ± SD), % predicted^b^	48.73 ± 18.41	97.98 ± 11.32	47.63 ± 15.62	107.83 ± 13.56	28.12 ± 7.38	99.97 ± 13.20	50.63 ± 17.44	94.91 ± 9.24
Sex (% male)	49.5%	50.1%	67.0%	57.9%	63.8%	100%	60.1%	50.1%

^a^Number packs of cigarettes smoked per day × number of years of smoking.

^b^Lung function was measured as forced expiratory volume in 1 second (*FEV*_1_) as a percentage of the predicted value.

### Gene variants and genotyping

SNP selection was based on previous genetic associations with disease and function (Table 3). The *NOS1* SNP (rs41279104) is located in the exon 1c regulatory region. Both the *NOS2* SNP (rs8078340) and the *NOS3* SNP (rs1800779) are located in their respective promoter regions. DNA from blood samples of the LHS participants was whole genome amplified using the REPLI-g Mini Kit (Qiagen, Mississauga, ON, Canada) prior to genotyping. The following TaqMan assays (Applied Biosystems, Foster City, CA, USA) were used: assay ID for rs41279104 = C__86363451_10; rs8078340 = C__29024700_10; rs1800779 = C__7599687_1. For each assay, 5 ng of DNA was used for allelic discrimination. For genome-wide SNP genotyping in the COPDGene, ECLIPSE, NETT-NAS, and GenKOLS populations, all cohorts were genotyped on Illumina platforms (Human Hap550, Quad610, and Omni1 Quad). Quality control in each cohort included subject missingness, discordances, relatedness, and sex; and marker missingness, discordances, singletons, and Hardy-Weinberg equilibrium.

**Table 3:** Description of the polymorphisms studied

**Gene**	**SNP**	**Protein**	**Allele change**	**Location**	**Association**
*NOS1*	rs41279104	Neuronal NOS	C = > T	Promoter for exon 1c	Reduced gene expression [12]
*NOS2*	rs8078340	Inducible NOS	G = > A	Promoter region	Decreased DNA-protein complex [13]
*NOS3*	rs1800779	Endothelial NOS	A = > G	Intronic	Lower FEV_1 _% predicted in COPD patients [14]

### Quantitative polymerase chain reaction (PCR)

RNA was extracted from lung tissue samples using the RNeasy Mini Kit (Qiagen) and cDNA was synthesized using SuperScript^®^III Reverse Transcriptase (Life Technologies, Grand Island, NY, USA). The cDNA samples were used to determine the gene expression of *NOS3*. The reference gene used was *GNB2L1* as this was previously shown to be stably expressed in lung tissue [22]. Gene expression assays for *NOS3* (Hs01574659_m1) and *GNB2L1* (Hs00272002_m1) were purchased from Applied Biosystems. Gene expression was calculated using cycle threshold (CT) values for *NOS3* and *GNB2L1*, as previously described [23].

### Protein expression levels

Human lung tissue fragments (30 mg) were homogenized in protein extraction buffer with protease and phosphatase inhibitors (Sigma-Aldrich, St. Louis, MO, USA). Protein lysates were resolved by SDS-PAGE and transferred to nitrocellulose membranes, and probed with anti NOS3 rabbit polyclonal antibody NOS3 (C-20): sc-654 at a concentration of 200 μg/mL (Santa Cruz Biotechnology, Inc., Santa Cruz, CA, USA) and anti-β-tubulin monoclonal antibody clone AA2 at a concentration of 1 mg/mL (Upstate Co., Lake Placid, NY, USA). Detection was performed with IR700 and IR800 anti-mouse and anti-rabbit antibodies (Cell Signaling Technology, Danvers, MA, USA). The density of the bands was analyzed with the Odyssey Infrared Imaging System (LI-COR Biotechnology, Lincoln, NE, USA) using two infrared channels independently. The results were expressed as NOS3 / β-tubulin density ratios.

### Immunohistochemistry

Sections were de-paraffinized, rehydrated, and antigen retrieved by autoclaving (15 min, 120°C, 30 psi) for 20 min in citrate target retrieval solution (Dako, Mississauga, ON, Canada). Endogenous peroxidase was quenched with 3% H_2_O_2_ and non-specific interactions blocked for 20 min with 10% goat serum. Antibody directed against human NOS3 (NOS3 (C-20): sc-654, Santa Cruz Biotechnology, Inc.) at 200 μg/mL was added overnight at 4°C in 5% goat serum. Sections were then incubated with biotinylated goat anti-mouse (1:100, Vector Labs Burlingame, CA, USA) for 60 min followed by a 10 min treatment with Streptavidin-HRP (Dako). The NOS3 antigen was visualized using the brown chromogen 3, 3-diaminobenzidine (Dako) and counterstained with Harris Hematoxylin Solution (Sigma-Aldrich). Finally, sections were then dehydrated and mounted with Cytoseal 60 (Richard-Allan Scientific, Kalamazoo, MI, USA). Antibody dilutions and all washes were in TRIS-buffered saline solution.

### Statistical analyses

The JMP 5.1 statistical software package (SAS Institute Inc., Cary, NC, USA) was used for analysis of the relationship between the genetic variants and the measures of lung function in the LHS. Agreement of the genotype distributions with Hardy-Weinberg equilibrium was assessed using a X^2^ goodness-of-fit analysis. The two outcomes used in the LHS were baseline and rate of decline of post-bronchodilator FEV_1_, expressed as a percent of predicted value. Statistical analyses were performed by multiple linear regression. For the COPDGene, ECLIPSE, NETT-NAS, and GenKOLS, after removal of principal component outliers, genotype imputation within each study was performed using MaCH and CEU samples from HapMap2 and the 1000 Genomes Project as a reference population. Association analysis of SNPs with case–control status was performed in each cohort using logistic regression, adjusting for age, pack-years of cigarette smoking, and genetic ancestry using PLINK 1.07. Results were combined among the four cohorts using fixed effect meta-analyses using METAL and R 2.12 (manuscript submitted). Differences in gene expression and protein levels between the three genotypes were assessed using two-tailed t-tests.

## Results

### Allelic discrimination in the lung health study

None of the three SNPs were in Hardy-Weinberg equilibrium for their genotypic distributions in the Caucasian LHS population (rs41279104, p-value = 0.04; rs8078340, p-value = 0.008; rs1800779, p = 0.003). We did not detect any association of either the rs41279104 or the rs8078340 polymorphisms with baseline FEV_1_ or rate of decline of FEV_1_ (Table 4). However, there was an association of the rs1800779 SNP with baseline lung function (p = 0.0018) (Table 4). This result remained significant (p = 0.0108) after Bonferroni correction for multiple comparisons (3 SNPs and 2 outcomes). In particular, subjects with the GG genotype had higher baseline FEV_1_ compared with subjects who had the AA genotype (p = 0.0273). Furthermore, with the AG + GG genotypes combined, an association with an increase in baseline FEV_1_ was observed compared with the AA genotype (p = 0.0042). No significant association, however, was observed with rate of decline of FEV_1_. An association study of this SNP was then performed in the four replication case–control populations; however no significant findings were observed (Table 5). In addition, we examined the relationship of rs1800779 to FEV_1_ % predicted in the four replication populations but there was no significant association with lung function in either the cases or the controls (Table 6).

**Table 4:** Genotype frequencies of NOS polymorphisms among participants in the LHS cohort and their associations with lung function

**Gene**	**SNP**	**Genotype**	**N**	**Mean** **±** **SE Baseline FEV**_**1 **_**(% predicted)**	p-value*^a^	p-value*^b^	**N**	**Mean** **±** **SE Rate of Decline in FEV**_**1 **_**(% predicted)**	p-value*^a^	p-value*^b^
*NOS1*	rs41279104	CC	2650 (79%)	78.39 ± 0.17	Referent	0.1424	2602 (79%)	-0.97 ± 0.04	Referent	0.3530
		CT	650(19%)	78.84 ± 0.37	0.7688		635 (19%)	-1.02 ± 0.07	0.5332	
		TT	55 (2%)	79.33 ± 1.19	0.3825		52 (2%)	-1.27 ± 0.26	0.3434	
*NOS2*	rs8078340	GG	2760 (76%)	78.64 ± 0.17	Referent	0.1118	2705 (76%)	-0.96 ± 0.03	Referent	0.1838
		AG	794 (22%)	78.28 ± 0.32	0.7166		781 (22%)	-1.03 ± 0.07	0.8221	
		AA	81 (2%)	77.82 ± 1.00	0.2977		81 (2%)	-1.12 ± 0.20	0.4215	
*NOS3*	rs1800779	AA	1448 (41%)	77.97 ± 0.24	Referent	**0.0018**	1425 (41%)	-1.05 ± 0.05	Referent	0.3838
		AG	1552 (44%)	78.81 ± 0.23	0.8034		1521 (44%)	-0.92 ± 0.05	0.1149	
		GG	516 (15%)	79.31 ± 0.39	**0.0273**		504 (15%)	-1.02 ± 0.07	0.7133	

*p-values were adjusted for age, sex, and smoking history (pack-years).

^a^p-value for comparison to the wild type homozygous genotype.

^b^p-value for the additive model.

**Table 5:** **Genetic association of *NOS3 *** **rs1800779 SNP with COPD affection status in each case**–**control study**

**Genotype**	COPDGene β coefficient (SE) p-value*	ECLIPSE β coefficient (SE) p-value*	NETT/NAS β coefficient (SE) p-value*	GenKOLS β coefficient (SE) p-value*	Meta-analysis β coefficient (SE) p-value*
AA	Referent	Referent	Referent	Referent	Referent
AG	0.03 (0.15) 0.83	0.01 (0.19) 0.95	0.36 (0.19) 0.06	0.03 (0.13) 0.81	0.08 (0.08) 0.30
GG	0.15 (0.22) 0.49	0.13 (0.28) 0.6310	0.10 (0.27) 0.70	-0.05 (0.19) 0.79	0.07 (0.12) 0.57

*p values were adjusted for age, pack-years of cigarette smoking, and genetic ancestry.

**Table 6:** **Genetic association of *NOS3 *** **rs1800779 SNP with FEV**_**1**_ % **predicted in each case**–**control study**

**Group**	**Genotype**	COPDGene β coefficient (SE) p-value*	ECLIPSE β coefficient (SE) p-value*	NETT/NAS β coefficient (SE) p-value*	GenKOLS β coefficient (SE) p-value*	**Meta**-analysis β coefficient (SE) p-value*
Cases	AA	Referent	Referent	Referent	Referent	Referent
	AG	0.75 (1.84) 0.68	-1.43 (0.85) 0.09	-1.46 (0.84) 0.08	1.50 (1.30) 0.25	0.80 (0.52) 0.12
	GG	2.08 (2.60) 0.42	-0.52 (1.23) 0.67	-1.42 (1.17) 0.23	-2.03 (2.04) 0.32	-0.88 (0.75) 0.24
Controls	AA	Referent	Referent	Referent	Referent	Referent
	AG	1.69 (1.10) 0.12	3.53 (2.35) 0.13	-0.49 (1.37) 0.72	-0.98 (0.68) 0.15	-0.09 (0.52) 0.86
	GG	-0.73 (1.64) 0.66	0.36 (3.47) 0.92	-1.62 (1.81) 0.37	-0.12 (1.05) 0.91	-0.51 (0.78) 0.51

*p values were adjusted for age, pack-years of cigarette smoking, and genetic ancestry.

### *NOS3* mRNA expression in lung tissue

**Figure 1:**
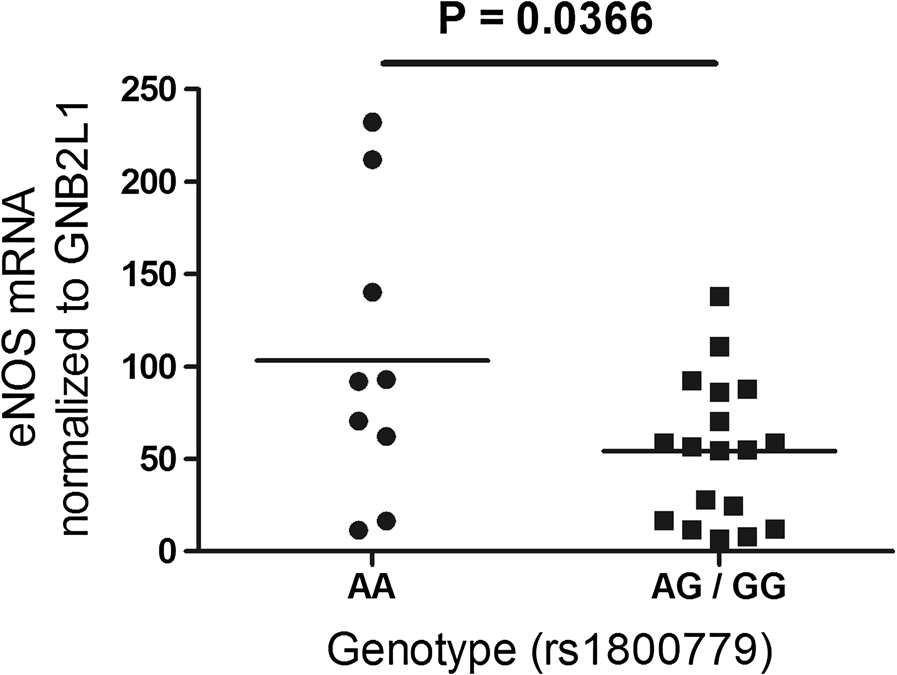
**The effect of rs1800779 on *NOS3 *** **mRNA expression in lung tissue.**

Since we had observed an association between the *NOS3* rs1800779 polymorphism and lung function levels in the LHS, a follow-up experiment was performed to determine the effect of rs1800779 on *NOS3* gene expression in lung tissue. Individuals were closely matched for age and sex among each genotype (7 males and 2 females for each genotype; average age 60 ± 6 years in all 3 groups). Of the subjects where smoking status was known, they were either ex-smokers (n = 12) or current smokers (n = 9). No association with *NOS3* gene expression was observed when all three genotypes were analyzed. However, as shown in Figure 1, subjects with the AG + GG genotypes combined demonstrated significantly lower *NOS3* gene expression in comparison with the AA genotype (p = 0.0366).

### NOS3 protein levels in lung tissue

**Figure 2:**
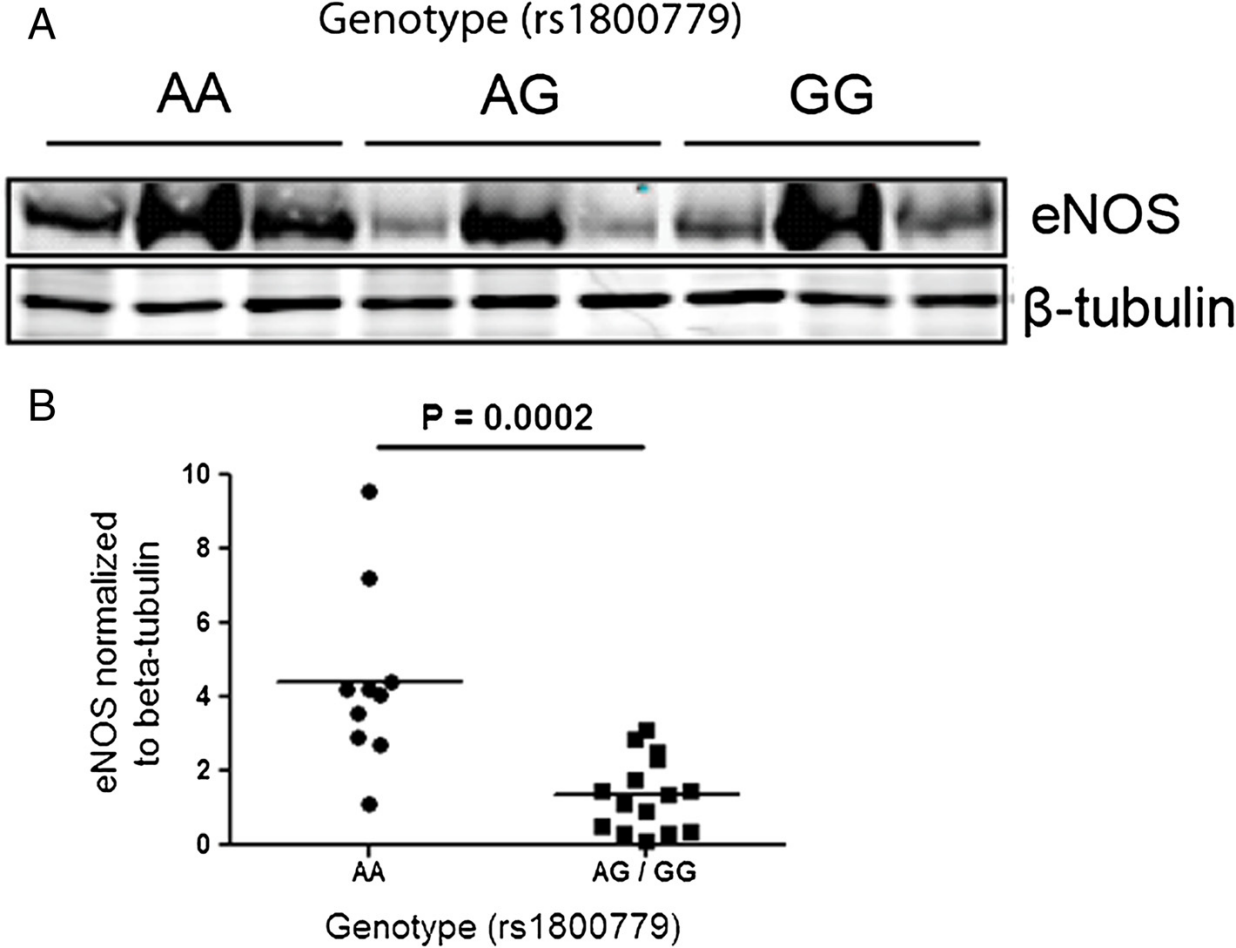
**The effect of rs1800779 on NOS3 protein levels in lung tissue. A**) Representative western blots of NOS3 protein levels in lung tissue using 3 randomly selected subjects from each genotype. **B**) NOS3 protein levels normalized to β-tubulin in different rs1800779 genotypic groups.

**Figure 3:**
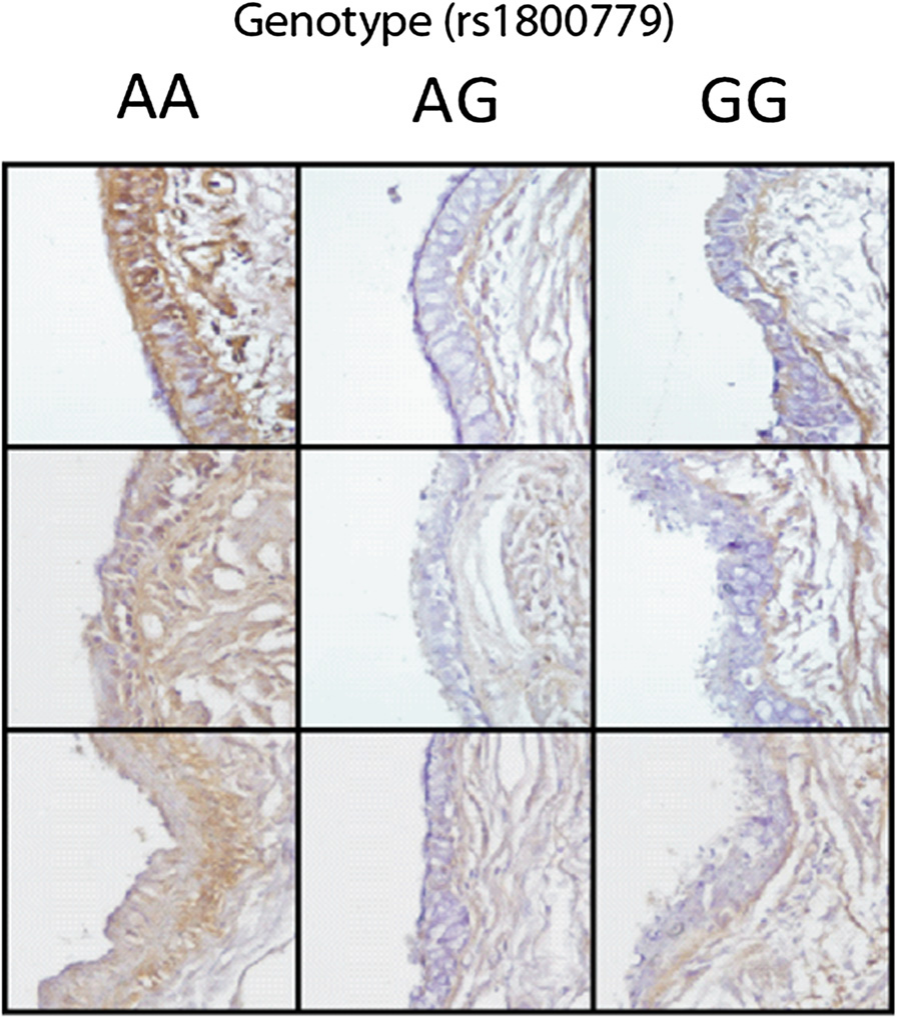
**NOS3 expression in the epithelium of lung tissue from subjects stratified by rs1800779 genotype (NOS3 positive staining denoted in brown).**

The same lung tissue samples used for *NOS3* mRNA expression were subsequently utilized for protein analysis by western blot. As shown in Figure 2, subjects with the rs1800779 AG + GG genotypes combined demonstrated significantly lower levels of NOS3 in comparison with individuals who had the AA genotype (p = 0.0002). We then used immunohistochemical analysis of lung tissue from three randomly selected donors of each genotype to localize NOS3 expression. As shown in Figure 3, the staining demonstrated NOS3 protein expression predominantly within the airway epithelium and again subjects with AA genotype demonstrated higher NOS3 expression compared with AG and GG individuals.

## Discussion

In this study we investigated three polymorphisms in the NOS genes in relation to cross-sectional lung function and rate of decline of lung function in COPD patients. Although we observed a significant association with rs1800779 in *NOS3* with baseline lung function in the derivation cohort we were unable to replicate the association in additional patient groups. Nevertheless, we were able to demonstrate that the rs1800779 SNP has a functional effect on the expression of the *NOS3* gene and this has implications for the numerous disease states that have been associated with this polymorphism [24-27].

The NOS gene variants that we investigated were limited to those that had strong *a priori* evidence for involvement in regulation of gene expression or in a trait related to COPD. We utilized this approach to maximize power by limiting the number of comparisons that were made. If a tag SNP approach had been used, a total of 137 polymorphisms would have had to be genotyped (using a minor allele cut off of 1 %, an r^2^ cut off for linkage disequilibrium of 0.8 in the European population, and a region 10 kb up- and downstream of each gene). Thus, the correction for multiple comparisons would be substantially more severe and the power of the study greatly reduced. The rationale for the SNP selection is described below.

The *NOS1* rs41279104 polymorphism that was selected for this study was previously shown to be associated with reduced gene expression [12]. The minor (T) allele was associated with a 30 % reduction in expression in a reporter gene assay [12]. The minor (A) allele of the *NOS2* rs8078340 polymorphism was associated with considerably decreased affinity for nuclear protein(s) [13] suggesting that it has functional significance. We prioritized these polymorphisms for investigation in this study, as we reasoned that these functional effects could be relevant to a variety of traits, including COPD. The rs1800779 polymorphism in *NOS3* was associated with COPD status and lower FEV_1_ % predicted in COPD patients [14]. This is the only published report of a NOS polymorphism associated with our disease of interest in the Caucasian population.

The rs1800779 polymorphism has been previously associated with a number of additional phenotypes but only one of these traits, cytokine responses in children at risk for asthma, was related to respiratory disease [24]. rs1800779 is in strong LD (r^2^ > 0.8) with ten other polymorphisms but none of these have been associated with phenotypes related to lung function or pulmonary disease.

Other NOS polymorphisms have been investigated with respect to phenotypes related to COPD. Arif *et al*. [14] studied four *NOS3* polymorphisms in north Indian COPD patients and controls: -786 T/C (rs3918161), -922A/G (rs1800779), 894G/T (rs1799983), and the 4B/4A variable number of tandem repeats (VNTR). rs3918161, rs1800779 and the VNTR were associated with COPD. However, data from the HapMap project (http://hapmap.ncbi.nlm.nih.gov/) show that rs3918161 is not polymorphic in individuals of European descent. It was not feasible to genotype the VNTR with the large sample sizes in this study and therefore rs1800779 was the only relevant polymorphism in our study populations.

Ahsan *et al*. investigated *NOS3* rs1799983 in 27 COPD patients and 66 controls but there was no significant difference (p = 0.18) in genotype frequency between the groups [28]. An earlier study that examined the *NOS3* VNTR did not find an association with COPD although there was an association with pulmonary hypertension in the patients [29]. Novoradovsky and colleagues genotyped six *NOS3* SNPs in patients with α_1_-antitrypsin deficiency and found that rs1799983 and rs1549758 were increased in severely affected cases compared with healthy controls [30]. However, these associations were not confirmed by a subsequent study of α_1_-antitrypsin deficient patients [31] and none of our patients were α_1_-antitrypsin deficient. *NOS2* polymorphisms have been investigated in the context of lung function growth and childhood asthma [32]. These investigators included 24 SNPs in their analysis - seven of which were in the *NOS2* promoter. The haplotype block including the promoter SNPs was associated with incident asthma and impaired lung function growth during adolescence. However, as the associations were seen in children and were with asthma, the relevance to COPD, a disease affecting an older demographic with a different pathology, is not clear. Several studies have examined the relationship between NOS gene variants and the fraction of exhaled NO in asthmatics and/or healthy subjects but the results have not been consistent [33-40] and therefore we did not consider them in this study.

The *NOS1* and *NOS2* polymorphisms that we investigated were both associated with functional effects on their respective gene expression [12,13]. Therefore, we hypothesized that these variants could be important factors in COPD, and that an association of these variants with FEV_1_ would be observed in smokers. However, we found no significant associations between the *NOS1* and *NOS2* polymorphisms and lung function in COPD patients.

The rs1800779 polymorphism in the promoter of *NOS3* was associated with lung function in the LHS participants. This SNP was not in Hardy-Weinberg equilibrium; however this could be due to several factors including random chance, genotyping assay failure, population stratification or because the SNP has a true genetic effect. In this study, it is unlikely a genotyping assay failure occurred since the positive controls were in 100% concordance with genotypes from the HapMap database (n = 73). The HapMap genotypes were generated with different technologies than the one used in this study and therefore the concordance is strongly indicative that the genotypes are accurate. Furthermore, approximately 13% of the subjects were re-genotyped and were in 100% concordance with the initial genotype results (n = 573). In addition, the LHS subjects were selected based on the presence of mild/moderate COPD and therefore the testing for Hardy-Weinberg equilibrium in this cohort may not be appropriate.

The results of the study in the LHS cohort demonstrated that the rs1800779 G allele was associated with a higher baseline FEV_1_. Although we found an association in the LHS we did not find any association of this SNP with COPD in four case–control populations. This may indicate that the association in the LHS is a false positive result, even though a limited number of polymorphisms were tested. Alternatively, the lack of replication may be a reflection of the different recruitment strategies and hence demographic factors in the cohorts involved e.g. the LHS participants were younger and had less severe airflow obstruction than the subjects in the other cohorts. The data presented in this study are contradictory to results in a previous paper which reported that the G allele of the SNP is associated with reduced lung function in COPD [14]. A reason for this discrepancy could be differences in the study populations. The cohort used in the previous study was composed of Indian subjects, whereas the cohort in this study only included Caucasian subjects.

A follow-up experiment was performed to determine the effect of the rs1800779 SNP on *NOS3* gene expression in lung tissue. It was observed that subjects who had the AG + GG genotypes had lower *NOS3* gene and protein expression compared with the AA genotype. In addition, immunohistochemical analysis demonstrated higher *NOS3* expression in the airway epithelium of COPD patients with the AA genotype. Taken together, these results strongly suggest that the G allele is associated with decreased *NOS3* expression.

The function of NO as a deleterious pro-inflammatory or protective anti-inflammatory agent has yet to be fully understood. However, there is evidence that NO is implicated in the pathogenesis of lung diseases such as COPD. NO is a radical molecule that can rapidly react with superoxides, yielding a cytotoxic molecule known as peroxynitrite. This compound has been shown to be an important factor contributing to tissue damage in chronic inflammation, as well as impairing key cellular functions [41]. In particular, nitrosative stress mediated by peroxynitrite is evident in patients with COPD, suggesting the toxic compound is a key contributor to the pathogenesis of the disease [7]. Therefore, it can be speculated that once exposed to an environmental pollutant, such as cigarette smoke, airway cells and tissues experience oxidative and nitrosative stress due to production of endogenous NO.

## Conclusions

Variants in the NOS genes were not associated with lung function or COPD status. However, the G allele of rs1800779 resulted in a decrease of *NOS3* gene expression and protein levels and this has implications for the numerous disease states that have been associated with this polymorphism. The rs1800779 polymorphism is also an excellent candidate for traits that are influenced by NO levels.

## Competing interests

The authors declare that they have no competing interests.

## Authors’ contributions

FA carried out the genotyping assays, participated in the statistical analysis and drafted the manuscript. FA, TLH, PDP, and AJS participated in the design of the Lung Health Study and gene expression sections. FA, DS and AS performed the quantitative PCR, western blotting and immunohistochemistry. MHC performed the statistical analysis for the replication cohorts. AG, PB, AL, DAL, WHA, THB and EKS participated in study design, recruiting, and sample collection for the replication cohorts. All authors read and approved the final manuscript.

## Pre-publication history

The pre-publication history for this paper can be accessed here:

http://www.biomedcentral.com/1471-2466/13/64/prepub
